# Improved Culture Medium (TiKa) for *Mycobacterium avium* Subspecies *Paratuberculosis* (MAP) Matches qPCR Sensitivity and Reveals Significant Proportions of Non-viable MAP in Lymphoid Tissue of Vaccinated MAP Challenged Animals

**DOI:** 10.3389/fmicb.2016.02112

**Published:** 2017-01-04

**Authors:** Tim J. Bull, Tulika Munshi, Heidi Mikkelsen, Sofie B. Hartmann, Maria R. Sørensen, Joanna S. Garcia, Paula M. Lopez-Perez, Sven Hofmann, Kai Hilpert, Gregers Jungersen

**Affiliations:** ^1^Institute of Infection and Immunity, St George’s University of LondonLondon, UK; ^2^National Veterinary Institute, Technical University of Denmark (DTU)Kongens Lyngby, Denmark

**Keywords:** *Mycobacterium avium* subspecies *paratuberculosis*, improved culture, quantification, qPCR, TiKa culture

## Abstract

The quantitative detection of viable pathogen load is an important tool in determining the degree of infection in animals and contamination of foodstuffs. Current conventional culture methods are limited in their ability to determine these levels in *Mycobacterium avium* subspecies *paratuberculosis* (MAP) due to slow growth, clumping and low recoverability issues. The principle goal of this study was to evaluate a novel culturing process (TiKa) with unique ability to stimulate MAP growth from low sample loads and dilutions. We demonstrate it was able to stimulate a mean 29-fold increase in recoverability and an improved sensitivity of up to three logs when compared with conventional culture. Using TiKa culture, MAP clumping was minimal and produced visible colonies in half the time required by standard culture methods. Parallel quantitative evaluation of the TiKa culture approach and qPCR on MAP loads in tissue and gut mucosal samples from a MAP vaccine-challenge study, showed good correlations between colony counts (cfu) and qPCR derived genome equivalents (Geq) over a large range of loads with a 30% greater sensitivity for TiKa culture approach at low loads (two logs). Furthermore, the relative fold changes in Geq and cfu from the TiKa culture approach suggests that non-mucosal tissue loads from MAP infected animals contained a reduced proportion of non-viable MAP (mean 19-fold) which was reduced significantly further (mean 190-fold) in vaccinated “reactor” calves. This study shows TiKa culture equates well with qPCR and provides important evidence that accuracy in estimating viable MAP load using DNA tests alone may vary significantly between samples of mucosal and lymphatic origin.

## Introduction

*Mycobacterium avium*subspecies *paratuberculosis* (MAP) is an economically important pathogen (McAloon et al., 2016) causing Johne’s disease in wide range of wild and domestic animals that has been linked as a zoonotic agent involved in the progression of Crohn’s disease in humans (Gitlin et al., 2012). The ability of MAP to exist in a variety of phenotypes, some with a high resistance to killing (Grant and Rowe, 2004), has increased the importance of providing accurate quantitative estimates of viable counts when testing for the presence of this pathogen in food (Botsaris et al., 2016; Galiero et al., 2016; Ricchi et al., 2016), animal and human samples (Timms et al., 2016). MAP is widely accepted as a difficult organism to culture reproducibly and accurately, particularly at low loads (Hines et al., 2007). This is particularly relevant in early stages of MAP disease pathogenesis which are often interspersed with periods of low MAP shedding, presumably as a result of diminutive loads in tissues (Kalis et al., 2001). MAP culture requires specialist media supplements, grows only relatively slowly, aggregates during liquid phases of sample preparation forming various sized colonies and like other pathogenic mycobacteria is difficult to recover when plated on solid media in very low dilution (Harris and Barletta, 2001; Elguezabal et al., 2011). It is particularly adapted to intracellular persistence and is known to exhibit several phenotypes (Nazareth et al., 2015). Whilst detection is not necessarily indicative of clinical disease, identifying the presence and quantity of viable MAP provides an important marker of disease and infectious spreading potential, particularly relevant to optimizing strategies of disease control at herd level and for individual assessments of treatment efficacies.

Quantitative detection of viable MAP by culture, particularly at low loads or from clinical tissue is thus considered challenging and as a consequence, molecular based detection systems have been developed (Whittington, 2009). To validate molecular methods, such as qPCR, however, requires calibration that assumes accurate colony forming unit or viability count estimations, efficient DNA extraction and DNA purification processing (Bull et al., 2003; Elguezabal et al., 2011). Robust techniques for DNA extraction and purification have provided means to specifically and reliably detect at least 100–500 genome equivalents (Geq) which when applied to adequate sample sizes can reduce the sensitivity to single log loads (Plain et al., 2014). The lack of culture correlation data, however, has until now prevented accurate means of estimating the true proportion of viable organisms in any sample tested.

In this study, we have evaluated a new culture process that uses supplements able to stimulate MAP growth. We show that for the first time, this generates sensitive, quantitative and reliable MAP recovery and culture from infected animals. Using this as a comparator with molecular methods we demonstrate that determining MAP presence by DNA based testing alone can significantly overestimate viable MAP presence in immune-reactive lymphatic tissue.

## Materials and Methods

### Media and Antibiotics

Middlebrook 7H9/7H11 media and OADC supplement were obtained from Becton Dickinson, UK, and Mueller Hinton broth from Merck, USA. All antibiotics were obtained from Apollo Scientific, UK and chemicals from Sigma, UK unless otherwise stated. All mycobacterial liquid cultures were set up in BACTEC MGIT 320 mycobacterial detection system which uses barcoded tubes with 7 ml media and additional growth supplement (Becton Dickinson, UK).

### Animals, MAP Challenge, and Vaccinations

Forty-nine Jersey calves (three heifers, 46 bulls) were enrolled in batches of seven on a Danish Jersey dairy farm with near zero prevalence of MAP infection as evidenced by several years of seroprevalence monitoring through the Danish Paratuberculosis eradication program. Following inoculation, all animals were housed in community pens with straw bedding in a secluded area of the farm. As animals reached 6 weeks old, they were randomly assigned to a single Silirum^®^ (CZ Veterinaria) (*N* = 1) immunization or two immunizations 4 weeks apart with one of two test vaccines (*N* = 2 + 2), or a saline sham-vaccination (*N* = 2).

All calves were MAP challenged 3 weeks after the last vaccination which was administered as three consecutive oral doses. The MAP challenge strain used was a clinical isolate Ejlskov2007 isolated from the feces of a disease cow cultured in liquid Middlebrook 7H9 supplemented with 10% OADC, 0.05% Tween 80 and 2 mg/ml Mycobactin J (MB7H9). A pool of Batch 190115 (7 g pelleted weight) and Batch 100315 (9.7 g pelleted weight) was thoroughly mixed and re-suspended in 120 ml of MB7H9 media with 15% glycerol before aliquoting into 5 ml vials (each containing 0.7 g pelleted MAP weight). To best estimate the number of MAP Geq per aliquot we used a qPCR directed against a single copy MAP gene (FadE5) using primer pairs (5′-AAGTCGAACAGGAACTTGGG-3′, 5′-TCGAGAACATCTTCCACCTG-3′) that had been previously shown to give accurate evaluations at the concentrations expected. All samples were run in duplicate using 2.5 μl DNA (5× pre-diluted) template, 12.5 μl QuantiTect SYBR green PCR kit (Qiagen, UK) and 0.125 μl of each primer (10 μM stock) in a total volume of 25 μl using a Rotor-Gene Q (Qiagen, UK) PCR machine. PCR cycling included an initial denaturation at 95°C for 15 min, followed by 45 cycles of 95°C: 30 s, 62°C : 60 s. Data analysis was performed using the Rotor-Gene Q Series Software version 1.7. All aliquots were then frozen at -80°C. One to three days prior to each block-inoculation, a vial was thawed, added to 15 ml sterile PBS, blended with single use sterile plastic homogenizer to resolve MAP clumps and refrigerated until use. On the morning of inoculation the material was re-suspended and aliquoted in 2 ml subsamples each containing 100 mg MAP. At the time of inoculation each tube was re-suspended in 800 ml warmed (37°C) fresh milk and fed individually by calf drench. A total of three inoculations were given to each calf every second day. All calves were fed milk daily up to the time of inoculation to maintain the gastro-oesophageal reflex bypassing the rumen.

### Sample Testing

At 28 weeks post challenge, all animals were euthanized and necropsied. Approximately 8 cm intestinal tissue was collected from ileal and jejunal sites of each animal located at various distances 0, 25, 50, 150, and 250 cm from the ileocecal valve in the proximal direction. All samples included Peyer’s patches were processed within 2 days of slaughter without prior freezing. DNA extraction method was as previous described (Park et al., 2014) with some modification. Samples of 100 mg tissue in 1.5 Milli-Q water were refrigerated 16–40 h and centrifuged at 15,000 × *g* for 15 min at room temperature (RT) and the tissue pellet re-suspended in 360 μl Qiagen tissue lysis (ATL) buffer and 40 μl proteinase K (Qiagen, UK) vortexed and incubated while shaking at 56°C for 1 h. Samples were again centrifuged 15,000 × *g* for 15 min (RT) and the supernatant discarded. Pellets were re-suspended in 275 μl enzymatic lysis buffer for gram-positive bacteria [20 mM TrisHCL (pH 8.0), 2 mM sodium EDTA, 1.2% Triton X-100, and 20 mg/ml lysozyme] and 200 μl 0.1 mm Zirconia/Silica beads (Biospec Products, Inc., USA) were added followed by incubation while shaking at 37°C for 30 min. This was followed by beat beating, 45 s at 30 rpm, using a TissueLyzer II (Retsch, Germany). To reduce foaming, samples were spun down for 30 s at 15,000 × *g*. Then 25 μl proteinase-K and 300 μl Qiagen DNeasy lysis (AL) buffer was added and incubated with gentle shaking at 56°C for 2 h followed by bead beating for 45 s at full speed (30 rpm) and centrifugation at 12,000 × *g* for 10 min. The supernatant was transferred to a new reaction tube without beads and 100% ethanol added at the ratio of 1 to 2 (ethanol:supernatant). DNA extraction procedure followed the Spin-Column protocol from the Qiagen DNeasy Blood and Tissue kit (Qiagen, UK). DNA was eluted in 50 μl AE buffer and frozen at -20°C. All samples were diluted five times and then only 1/10 of each sample was assayed to avoid PCR inhibition due to possible DNA overload.

Quantitative PCR was performed using an in house system (Thakur, 2012) validated against a MAP DNA/spiked tissue DNA calibration curve using reactions targeting IS*900* with the validated primer set 5′-GGCAAGACCGACGCCAAAGA-3′, 5′-GGGTCCGATCAGCCACCAGA-3′. IS*900* was used in preference to FadE5 due to its presence in multiple copies that ensure increased sensitivity and robustness of testing low loads. All samples were run in duplicate as above with denaturation at 95°C, followed by 45 cycles of 95°C: 30 s, 68°C: 60 s. Data analysis was performed using the Rotor-Gene Q Series Software version 1.7 using a calibration set of DNA dilutions from a standard MAP DNA stock included in all runs (efficiency: 89%, *R*^2^: 0.99780). The lower detection limit was determined according to a standard Cq of 33.57 representing a previously estimated sensitivity of approximately 1.7 Geq. Acceptable duplicate variation was set at 1.5 Cq.

### Culture

Hexadecylpyridinium chloride (HPC: Sigma, UK) sample decontamination was carried out according to World Organisation for Animal Health (OIE) standard protocols (Edwards, 2007). Briefly, 100 mg of homogenized sample was added to 1.5 ml sterile 0.75% HPC and incubated at 37°C for 3 h. Samples were centrifuged at 1,600 × *g* for 30 min, supernatant discarded, the pellet resuspended in 0.5 ml of ½ strength Muller Hinton (½MH) Broth with 100 μg/ml Vancomycin, 50 μg/ml Amphotericin B, 100 μg/ml Nalidixic acid and incubated overnight at 37°C with gentle shaking. Tubes were allowed to settle for 30 min and 100 μl inoculum taken from the middle of the suspension.

TiKa-Kic sample decontamination used 100 mg of homogenized sample digested for 5 h at 37°C with gentle shaking (200 rpm) in 1 ml CT digest buffer [8.5 mg/ml NaCl, 1 mg/ml CaCl_2_, 100 mg/ml Collagenase B (Roche, UK), and 100 mg/ml Trypsin]. Digests were centrifuged at 14,000 × *g* for 10 min and the supernatant discarded. The pellet was then re-suspended in 10 ml ½MH broth supplemented with TiKa-Kic (TiKa Diagnostics, UK) and incubated for 24 h at 37°C with gentle shaking. Samples were centrifuged 1,600 × *g* for 30 min and the pellet re-suspended in 600 μl sterile PBS.

TiKa14D-7H11 solid culture used Middlebrook 7H11, 10% OADC, 2 mg/ml Mycobactin J (ID-Vet, France), 25 μg/ml Vancomycin, 30 μg/ml Amphotericin B, 25 μg/ml Nalidixic acid, 1 μg/ml TiKa-14D and was inoculated with effectively 1/6 (100 μl) of each treated sample. All plates were read from 3 weeks and cfu’s confirmed at 10 weeks.

TiKa-MGIT liquid culture used 7 ml MGIT tubes with 0.8 ml growth supplement, 2 mg/ml Mycobactin J (ID-Vet, France), 25 μg/ml Vancomycin, 30 μg/ml Amphotericin B, 25 μg/ml Nalidixic acid, 1 μg/ml TiKa-14D and were inoculated with 5/6 (500 μl) of each treated sample. Sample loads were calculated by extrapolation using a calibration algorithm from Time to Positivity (TTP measured in days) of each MGIT tube when flagged positive by the Bactec MGIT 320 mycobacterial detection system as determined by the manufacturer. The calibration algorithm cfl/g tissue = (3 × 10^6^)^(-0.347×TTPdays)^ was determined from a previous spiking experiments of known dilutions of MAP (strain K10) grown in TiKa14D + MGIT media (data not shown). Processing by qPCR for IS*900* was performed in a separate research facility to culture and all results and identities of animal groups were kept blinded until the end of the experiment.

Samples from various tissue sites obtained from each animal at necropsy were processed in parallel to compare standard and TiKa-Kic methods. They were then quantitatively tested in parallel for the presence of either MAP Geq by qPCR, colony forming units (cfu) using TiKa14D + 7H11 colony counting or extrapolated colony forming load (cfl) by determining initial inoculum load from TTPdays results of TiKa14D + MGIT liquid culture. Samples from one cull set were additionally tested by MAP culture on unmodified 7H11 plates after preparation using a conventional HPC based method (cfu, HPC). Mean individual total counts for each of the methods (cfu/cfl/Geq) were determined by summing the estimated load from each of the 10 samples from each animal.

### Statistical Analysis

All statistical analyses were performed using Graph Pad Prism v6.01. For all analyses, a *p*-value of <0.05 was considered to be statistically significant. The criteria for interpreting the Spearman’s rank correlation with *p*-value <0.001 was >0.75 = excellent, <0.40 = poor, 0.40–0.75 = good (Fleiss et al., 2003).

## Results

Seven blocks of seven calves (*n* = 49) were purchased at 6 weeks of age and randomly assigned to vaccine or sham-vaccinated groups. Results of individual vaccine efficacy will be reported elsewhere. Two animals were euthanized as a result of causes unrelated to the experiment (one broken leg, one malformed pyloric sphincter) and were excluded. The remainder, comprising 34 vaccinated and 13 sham-vaccinated (Saline) individuals were successfully challenged 3 weeks post vaccination with an oral dose of MAP (total inoculum estimated by qPCR : 1 × 10^13^ Geq) and maintained in appropriate housing for 28 weeks post challenge. Final MAP loads adjusted for sample weight and collated as cfu, cfl, or Geq per 100 mg of sample tissue (**Supplementary Table S1**) showed all animals had at least 70% of samples positive for MAP by at least one method with 3% (16/470) being negative by all three methods. Sample contamination due to direct carry over of viable non-mycobacterial flora was seen in only one of 470 sample preparations using the TiKa-Kic with TiKa14D + MGIT liquid culture. No carry over contamination was seen using HPC with 7H11 or TiKa-Kic with TiKa14D + 7H11 culture.

**FIGURE 1:**
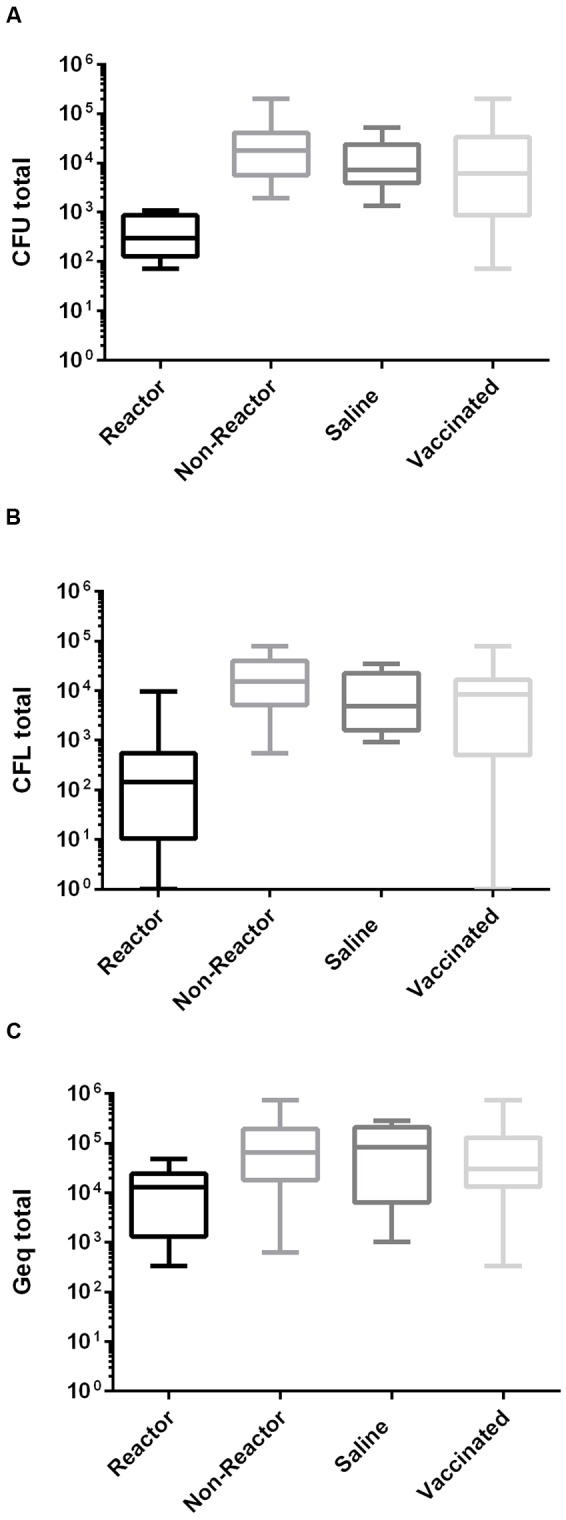
**Distribution plots of total *Mycobacterium avium* subspecies *Paratuberculosis* (MAP) loads in different methods.** Plots derived by summing loads from 10 samples for each animal determined from the same set of samples (*n* = 470) by three separate methods **(A)** cfu using TiKa-Kic/Tika14D + 7H11 method (*p* = <0.0001), **(B)** cfu using TiKa-Kic/Tika14D + MGIT method (*p* = <0.0002) and **(C)** Geq using qPCR (*p* = <0.042) on control and vaccinated groups of calves. There was no statistical significance between saline and non-reactor groups in all methods.

Comparing total load estimates of individual vaccinated animals with the sham-vaccinated (saline) control group we were able to identify a subgroup (designated as “reactor” group) within the vaccinated animal group that had total load means significantly (cfu: *p* = <0.0001; cfl: *p* = <0.0002; and Geq: *p* = 0.042) below that of controls by all three methods (**Figure 1**).

**FIGURE 2:**
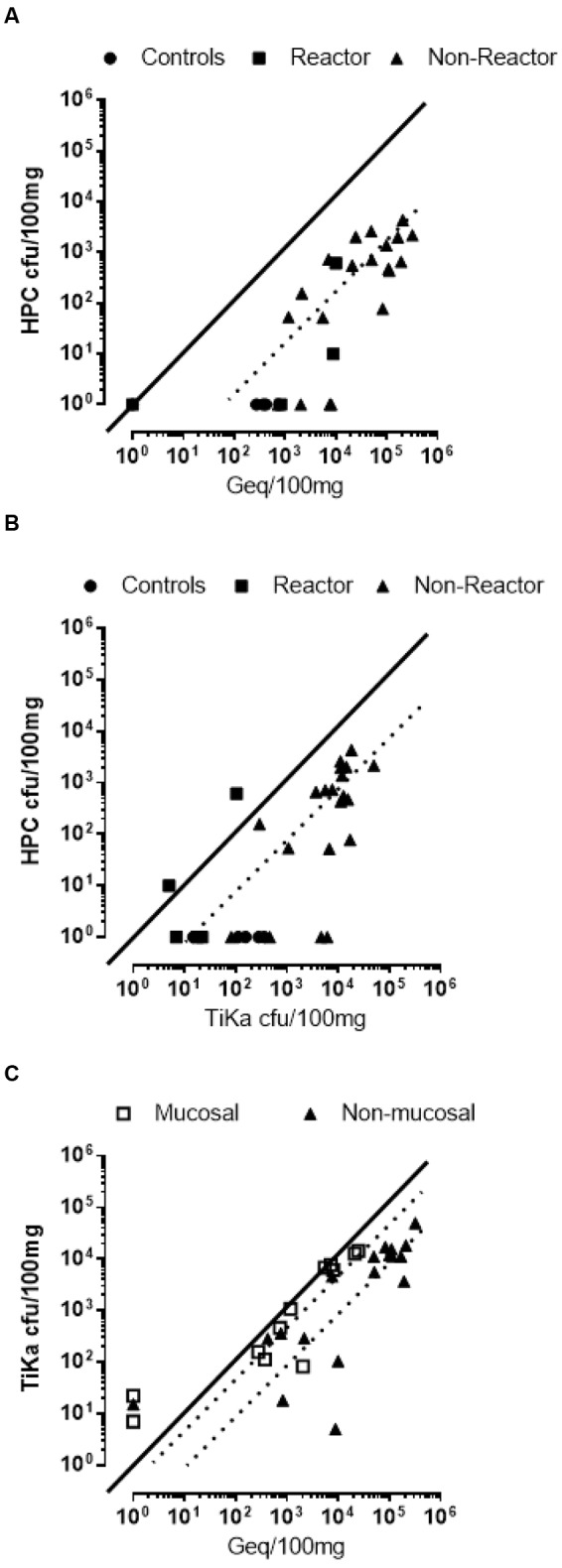
**Correlation plots of MAP loads in individual samples from mucosal and non-mucosal tissues.** These plots were estimated by three methods **(A)** cfu by hexadecylpyridinium chloride (HPC) with qPCR at median 72-fold, mean 189-fold, and Spearman *r* at 0.810; **(B)** cfu by HPC with cfu by TiKa-Kic/Tika14D + 7H11 at median eightfold, mean 29-fold, and Spearman *r* at 0.723 and **(C)** cfu by TiKa-Kic/Tika14D + MGIT with qPCR at median 1.6-fold, mean 13-fold, and Spearman *r* at 0.963 for mucosal samples and at median eightfold, mean 117-fold, and Spearman *r* at 0.845 for non-mucosal samples (qPCR = Geq). Dotted lines represent medians.

The conventional HPC + 7H11 solid method performed the poorest relative to all other methods. Cultures showed an apparently random proportion of large colonies indicative of clumping present in all HPC positive cultures and required a significantly longer period (TiKa-Kic with TiKa14D + 7H11 culture 3–4 weeks, HPC 8–10 weeks) to generate visible colonies. Contrastingly, nearly all colonies grown using TiKa-Kic with TiKa14D + 7H11 culture were of a regular size and not suggestive of growing from clumps. There was good correlation of load estimates (*r* = 0.810, *p* = <0.0001) between Geq from qPCR and cfu from HPC at high sample loads (**Figure 2A**) but this was shifted from a 1:1 relationship by a mean 189-fold (median 72-fold) difference. In addition the sensitivity of detection (negative cut off) for HPC treated sampling was relatively low with 37% (12/30) positive qPCR values showing no cfu on HPC with 7H11 solid medium even after 12 weeks incubation. In this small sample comparison the TiKa-Kic with TiKa14D + 7H11 protocol was markedly superior to HPC with conventional 7H11 solid medium. TiKa-Kic with TiKa14D + 7H11 culture provided a mean 29-fold (median eightfold) increase in cfu values relative to HPC with 7H11 culture (**Figure 2B**) and markedly improved recovery with MAP being grown from all HPC negatives (range 7–6,129 cfu/100 mg).

**FIGURE 3:**
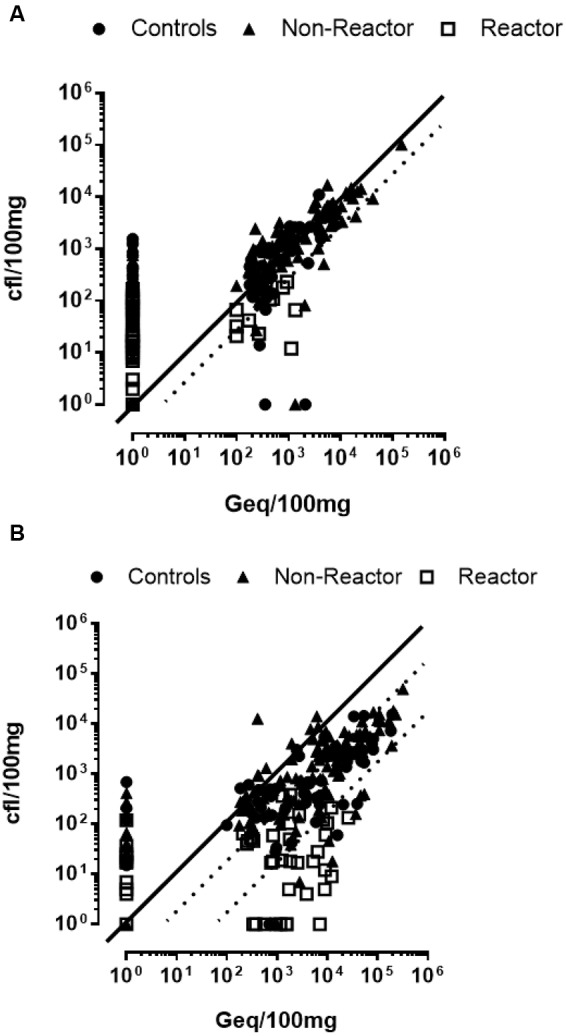
**Correlation plots of MAP loads comparing TiKa14D + 7H11 and qPCR.** Individual samples estimated as cfu by TiKa-Kic/Tika14D + 7H11 and Geq by qPCR for **(A)** mucosal having control (median onefold and mean twofold), vaccinated non-reactor (median twofold and mean twofold) and vaccinated reactor (median fourfold and mean 13-fold) groups of calves and **(B)** non-mucosal tissues also having control (median eightfold and mean 19-fold), vaccinated non-reactor (median eightfold and mean 29-fold) and vaccinated reactor (median 60-fold and mean 190-fold) groups of calves. Dotted lines represent medians. Spearman *r* for each was 0.723 and 0.717, respectively.

TiKa solid culture showed excellent correlation with qPCR, particularly in mucosal samples (*r* = 0.963, *p* = <0.0001) that approached a linear relationship (mean 13-fold, median 1.6-fold differences in count loads). When these data were separated into populations of mucosal and tissue samples the correlation remained excellent but reduced (*r* = 0.845, *p* = <0.0001) with fold differences in count loads shifting proportionally toward Geq (mean 117-fold, median eightfold), suggesting variations influencing cfu values could be tissue specific (**Figure 2C**). Extension of this analysis correlating qPCR Geq values and TiKa-Kic with TiKa14D + 7H11 cfu across all tested animals confirmed this observation (**Figure 3**). Correlation of Geq and cfu using TiKa-Kic with TiKa14D + 7H11 across all (*n* = 282) animal mucosal samples (**Figure 3A**) was good (*r* = 0.723, *p* = <0.0001) with a close linear relationship over a wide range of values (mean twofold, median onefold). A significant shift was again observed in mean differences between Geq and cfu in non-mucosal tissue samples with mean 19-fold (median eightfold) for controls that increased significantly to 232-fold (median 57-fold) if the vaccine “reactor” group was considered separately (**Figure 3B**). In this experiment, TiKa-Kic with TiKa14D + 7H11 culture was more sensitive than qPCR with 28% (79/282) samples cfu positive/qPCR Geq negative (range 2–156 cfu/100 mg tissue). In contrast only 1% (3/282) of mucosal samples were cfu negative/PCR Geq positive (range 36–209 Geq/100 mg tissue).

Correlations with qPCR and TiKa-Kic with TiKa14D + MGIT liquid culture had similar outcomes with near linear correlations in mucosal samples (**Supplementary Figure S1**) and a significant shift in mean differences of 107-fold (median 53-fold) in “reactor” animals relative to qPCR. TiKa-Kic with TiKa14D + MGIT liquid culture was also more sensitive than qPCR with 28% (79/282) samples cfl positive/qPCR Geq negative (range 2–156 cfl/100 mg tissue) and only 1% (3/282) of mucosal samples were cfl negative/qPCR Geq positive (range 36–209 Geq/100 mg tissue).

## Discussion

The slow nature of MAP growth and its consequent sample turnaround time with conventional culture has promoted the development of highly sensitive molecular methods as an attractive rapid alternative. However, molecular quantification of Geq as a measure does not provide any indication of load viability. Previous comparative studies of qPCR and conventional culture have demonstrated good correlation in high sample loads present in feces (Douarre et al., 2010; Mita et al., 2016) but the inability of conventional culture methods to accurately culture low loads of viable organisms from clinical samples introduces problems. Culture sensitivity for MAP has only ever been as good as 2–3 log_10_ (Ricchi et al., 2016), thus at low loads correlations to DNA presence are difficult to obtain and the true relationship between genome equivalent values and the demonstrable viable MAP count in these samples remains uncertain (Kralik et al., 2012; Plain et al., 2015). The underlying reasons for these discrepancies are probably multi-variant. DNA detection from difficult samples such as feces are often significantly influenced by carry-through of amplification enzyme inhibitors and the necessity for multiple steps in sample processing that can introduce error (Timms et al., 2015). Furthermore, the need to decontaminate samples of commensal bacterial and fungal flora introduces MAP exposure during sample preparation to chemicals and antibiotics that can inhibit and in some cases kill large proportions of the viable load (Gumber and Whittington, 2007; Kralik et al., 2014). These variables make it difficult to define the number of samples and quantity of any one sample which should be tested to gain significant confidence when assessing true test negativity.

In this study, we have used samples available from a vaccine-challenge experiment to evaluate the Tika culture system which uses a novel sample preparation protocol (TiKa-Kic) and supplemented growth media (TiKa + 7H11, TiKa + MGIT). The sample preparation method differs from conventional protocols in not requiring harsh chemical treatment of samples such as HPC, sodium hydroxide and oxalic acid to remove contaminating flora. The TiKa-Kic killing cocktail has no influence on mycobacterial growth or viability but is effective against a wide range of other bacterial and fungal genera. When followed by growth in conventional MAP media (either liquid or solid) supplemented with a growth enhancer (TiKa14D) there is stimulation of MAP growth and suppression of both MAP aggregation/clumping and entry into lag phase. Parallel processing of samples from experimentally challenged calves comparing TiKa with a standard HPC sample protocol showed a mean 184-fold greater growth of MAP load with colonies appearing 3–4 weeks before the majority of colonies detected using HPC treated samples. In addition 30% of qPCR positive processed samples when processed with conventional HPC treatment, produced no visible recovery after 8–10 weeks incubation whilst TiKa-Kic treatment allowed growth of regular sized colonies in each of these samples.

Parallel processing of mucosal tissue samples from experimentally infected calves showed that TiKa culture gave excellent correlations at medium and high MAP loads with genome equivalent (Geq) estimates derived using a qPCR with a dynamic range cut off of 1.7 Geq. A major finding of this study showed that TiKa culture was the most sensitive test with 22% of cfu positive TiKa treated samples from control animals being negative in qPCR (mean = 455 cfu : range 14–1,564 cfu). This difference could be explained to some extent by the specific sample volume used for qPCR which was required to be 20-fold less than for TiKa sampling to ensure avoidance of carry over inhibitors. These results suggest TiKa culture was able to consistently recover and grow colonies from a significant majority of the MAP load within mucosal tissue and that this represents a 2 log_10_ improvement over any existing culture protocol. Subsequent testing using this qPCR method should consider raising the sample volume if possible.

Further analysis of the data was able to discern that fold differences between MAP genome equivalent loads (qPCR) and MAP viable loads (cfu) in lymph node tissues was significantly and consistently different to that of mucosal samples. Correlations of cfu and Geq values from MAP infected control animals remained linear but produced a mean 19-fold (median eightfold) decrease in culturable MAP load of lymphatic tissue compared to mucosal tissue. There was no evidence of increases in clumping in these samples and the correlation appeared good over the whole range of loads suggesting that this was not a technical phenomenon. Similar shifts were observed in both solid and liquid TiKa supplemented media. We surmise that lymphatic tissue was evoking a significant effect on the viability of this proportion of MAP in all animals regardless of vaccination status. Importantly this was not the case in mucosal tissue suggesting that this effect was tissue specific. Interestingly, a sub group of the animals (referred to here as “reactors”) that had been MAP-vaccinated prior to MAP challenge and had responded by decreasing the MAP load in both mucosal and lymphatic tissue significantly below any of the sham-vaccinated controls showed the largest effect in this regard. Vaccine “reactors” had a mean 100-fold greater reduction in fold differences between Geq and cfu than seen in mucosal tissue from the same animals and 10-fold greater reduction than equivalent tissue from sham-vaccinated controls. This data suggests that at the selected time point (7 months post challenge) MAP vaccine “reactors” were harboring up to 3 log_10_ greater proportion of dead or possibly non-culturable/viable “dormant” MAP in their lymphatic tissue. Why this population should be absent from mucosal tissue requires more directed studies including detailing the predominant metabolic state and degree of viability of MAP in active lymphatic tissue. We hypothesize that vaccine “reactors” are generating more active MAP killing mechanisms and the increased residual DNA presence in lymphatic tissue is a result of the detection of killed MAP which unlike the mucosal compartment have not yet been fully processed and translocated for excretion.

## Conclusion

TiKa culture provides the most efficient and rapid method of culturing MAP so far described. It validates the use of qPCR for rapid determination of viable MAP load in mucosal tissue and when applied together with qPCR could offer the possibility of a novel method to monitor vaccine efficacy. Importantly this study shows that qPCR is not an accurate method of quantifying viable MAP load in lymphatic tissue as this varies widely within individuals with diverse immunologically reactive status. Future studies are required to evaluate the utility of TiKa culture and determine the true proportion of MAP viability in these samples.

## Ethics Statement

All animal procedures were approved and controlled by the Danish National Experiments Inspectorate.

## Author Contributions

GJ conceived and designed the experiments. TB co-ordinated culture experiments, analyzed data, and wrote the paper in collaboration with all authors. GJ, JG, SBH, HM, MS, and TM performed experiments and analyzed data. KH, PL-P, and SH contributed reagents and materials.

## Conflict of Interest Statement

TB and KH Hilpert declare their affiliation with TiKa Diagnostics Ltd. The company did not influence the design, conduction, interpretation, or evaluation of this study. All the other authors declare that the research was conducted in the absence of any commercial or financial relationships that could be construed as a potential conflict of interest.

## Supplementary Material

FIGURE S1 **Correlation plots of MAP loads comparing TiKa-MGIT and qPCR.** Individual samples estimated as cfu by TiKa-Kic/Tika14D + MGIT and Geq by qPCR for **(A)** mucosal having control (median onefold, mean onefold), vaccinated non-reactor (median onefold, mean twofold) and vaccinated reactor (median onefold, mean fivefold) groups of calves and **(B)** non-mucosal tissues also having control (median 30-fold, mean 61-fold), vaccinated non-reactor (median 21-fold, mean 39-fold) and vaccinated reactor (median 53-fold, mean 107-fold) groups of calves. Dotted lines represent medians. Spearman *r* for each was 0.744 and 0.681 respectively.

Table S1 **Results of observed cfu and calculated cfl, Geq obtained from each individual sample.** For clarity results are tabulated into three categories (Vaccinated ‘Reactors’, Vaccinated ‘Non-Reactors’ and Sham vaccinated Controls). Each sample code uses the format; animal number – tissue site (I = Ileum, J = Jejunum, IV = Ileocecal Valve, LN = Lymph node) – distance of sampling site along Ileum or Jejunum. Median and Mean values of each protocol are tabulated under the ‘Reactor’ group columns.
