# Supplementary figures and images for: Improved Culture Medium (TiKa) for Mycobacterium avium Subspecies Paratuberculosis (MAP) Matches qPCR Sensitivity and Reveals Significant Proportions of Non-viable MAP in Lymphoid Tissue of Vaccinated MAP Challenged Animals
Source: Front Microbiol. 2017 Jan 4;7:2112. doi: 10.3389/fmicb.2016.02112 (PMC5209360; doi:10.3389/fmicb.2016.02112)

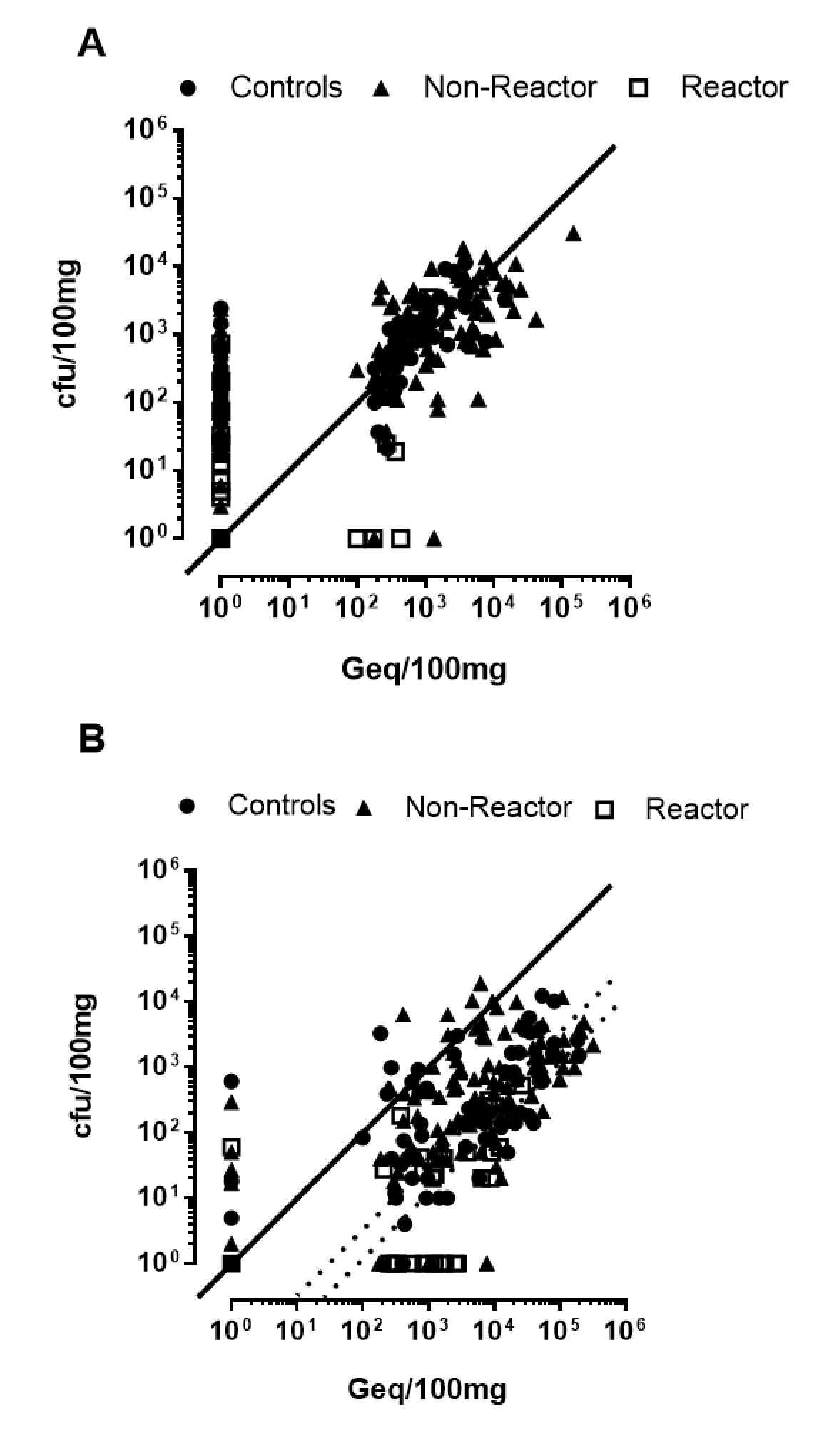

Supplement: FIGURE S1 — Correlation plots of MAP loads comparing TiKa-MGIT and qPCR. Individual samples estimated as cfu by TiKa-Kic/Tika14D + MGIT and Geq by qPCR for (A) mucosal having control (median onefold, mean onefold), vaccinated non-reactor (median onefold, mean twofold) and vaccinated reactor (median onefold, mean fivefold) groups of calves and (B) non-mucosal tissues also having control (median 30-fold, mean 61-fold), vaccinated non-reactor (median 21-fold, mean 39-fold) and vaccinated reactor (median 53-fold, mean 107-fold) groups of calves. Dotted lines represent medians. Spearman r for each was 0.744 and 0.681 respectively. [file Image_1.JPEG]
